# siRNADiscovery: a graph neural network for siRNA efficacy prediction via deep RNA sequence analysis

**DOI:** 10.1093/bib/bbae563

**Published:** 2024-11-06

**Authors:** Rongzhuo Long, Ziyu Guo, Da Han, Boxiang Liu, Xudong Yuan, Guangyong Chen, Pheng-Ann Heng, Liang Zhang

**Affiliations:** School of Basic Medicine and Clinical Pharmacy, China Pharmaceutical University, 211198, Nanjing, China; Department of Computer Science and Engineering, The Chinese University of Hong Kong, Central Ave, Hong Kong SAR, China; Institute of Molecular Medicine (IMM) and Department of Laboratory Medicine, Renji Hospital, School of Medicine, Shanghai Jiao Tong University, 200240, Shanghai, China; Hangzhou Institute of Medicine (HIM), Chinese Academy of Sciences, 310022, Hangzhou, Zhejiang, China; Department of Pharmacy, Faculty of Science, National University of Singapore, Singapore, 117543, Singapore; ACON Pharmaceuticals, 2557 Route 130 S, Ste 3, Cranbury, NJ 08512, USA; Zhejiang Lab, Ke Chuang Avenue, 311121, Zhejiang, China; Department of Computer Science and Engineering, The Chinese University of Hong Kong, Central Ave, Hong Kong SAR, China; Hangzhou Institute of Medicine (HIM), Chinese Academy of Sciences, 310022, Hangzhou, Zhejiang, China

**Keywords:** siRNA efficacy prediction, gene silencing efficacy, RNA sequence analysis, graph neural network, deep learning

## Abstract

The clinical adoption of small interfering RNAs (siRNAs) has prompted the development of various computational strategies for siRNA design, from traditional data analysis to advanced machine learning techniques. However, previous studies have inadequately considered the full complexity of the siRNA silencing mechanism, neglecting critical elements such as siRNA positioning on mRNA, RNA base-pairing probabilities, and RNA–AGO2 interactions, thereby limiting the insight and accuracy of existing models. Here, we introduce **siRNADiscovery**, a Graph Neural Network (GNN) framework that leverages both non-empirical and empirical rule-based features of siRNA and mRNA to effectively capture the complex dynamics of gene silencing. On multiple internal datasets, siRNADiscovery achieves *state-of-the-art* performance. Significantly, siRNADiscovery also outperforms existing methodologies in *in vitro* studies and on an externally validated dataset. Additionally, we develop a new data-splitting methodology that addresses the data leakage issue, a frequently overlooked problem in previous studies, ensuring the robustness and stability of our model under various experimental settings. Through rigorous testing, siRNADiscovery has demonstrated remarkable predictive accuracy and robustness, making significant contributions to the field of gene silencing. Furthermore, our approach to redefining data-splitting standards aims to set new benchmarks for future research in the domain of predictive biological modeling for siRNA.

## Introduction

RNA interference (RNAi) serves as a critical regulatory mechanism in cells, utilizing short double-stranded RNA (dsRNA) molecules to guide the homology-dependent regulation of gene expression [1]. This process involves small interfering RNAs (siRNAs), which are RNAi-based regulators consisting of 19-23 nucleotides. These siRNAs are processed from longer dsRNA precursors by Dicer, an RNase III-like enzyme, and then incorporated into the RNA-induced silencing complex (RISC). Within RISC, siRNA strands are separated, and the strand with a more stable 5’ end guides the complex to bind homologously to the target mRNA. The catalytic component of RISC, a member of the Argonaute family (AGO2), then cleaves the target mRNA, leading to its degradation and the silencing of the gene [2].

Thousands of potential siRNAs can target the same gene, thus, identifying the most effective siRNA from these candidates is currently a focus of study. Researchers have developed various algorithms to predict siRNA activity, categorized into **two** generations. The **first**-generation tools were based on empirical rules from validated small siRNA datasets [3], but lacked accuracy [4]. The limitations were attributed to the small dataset sizes that missed several crucial features [5]. With the expansion of siRNA data and the development of artificial intelligence, the **second**-generation algorithms have improved predictions of siRNA knockout efficiency by utilizing advanced data mining techniques, including s-Biopredsi [6], DSIR [7], and i-Score [8]. However, **two main issues** impair the performance of these early second-generation algorithms. ***Firstly***, the performance of these algorithms exhibits instability, potentially stemming from the heterogeneity of siRNA datasets, which are assessed by various groups across different protocols, and largely depend on feature selection [9]. ***Secondly***, the numerous interactive factors and nonlinear characteristics of biological processes pose significant challenges to the construction and optimization of traditional models [10].

To address the first issue, some advanced artificial intelligence models have been employed in the development of second-generation algorithms [11–13]. Han *et al.* [11] enhanced prediction models with a Convolutional Neural Network (CNN) that analyzed sequence context and thermodynamic properties, offering improved stability and efficiency. However, the CNN struggled to consider the second issue. Graph Neural Networks (GNNs) have been proven effective in handling complex data interactions [14], particularly in constructing predictive models for siRNA–mRNA networks based on mutual characteristics. Recently, Massimo *et al.* [12] introduced the first GNN-based model named GNN4siRNA for siRNA inhibition prediction, featuring three node types, siRNA, mRNA, and interaction nodes. However, GNN4siRNA used k-mers for sequence embedding to focus on local sequence patterns with multiple nucleotides, potentially overlooking or diminishing specific nucleotide positions. Additionally, inappropriate k-values may lead to the omission of crucial information or introduction of noise. Moreover, there are several key factors previously discussed that critically influence the efficacy of siRNA-mediated gene silencing ignored by GNN4siRNA, such as positional context of siRNA along the mRNA strand [15], base-pairing secondary structure [16], and empirical rules (i.e., nucleotide frequencies [17], G/C percentages [18], and impact of nucleotides at each position [19]), which undermines the performance of the model.

To this end, we propose **siRNADiscovery**, a GNN framework that thoroughly explores the sequence features of siRNA and mRNA with a specific topological structure, enhancing siRNA efficacy prediction performance. Our siRNADiscovery first extracts two distinct-types of RNA features, i.e., non-empirical features and empirical rule-based ones, and integrates them into GNN training. Specifically, the non-empirical features include one-hot sequence encoding, positional encoding, base-pairing probabilities, and RNA–protein interaction probabilities. The empirical rule-based features include the thermodynamic stability profile, nucleotide frequencies, the G/C percentages, and the rule codes in each position of siRNA. Compared with other existing methods, the experimental results on all datasets demonstrate that our model achieves *state-of-the-art* performance. Upon that, we notice a data leakage issue within the cross-validation setting, i.e., an mRNA from the test set might have been included in the training phase. We propose a new standard method for evaluation within the cross-validation setting, and siRNADiscovery shows stable performance under different splitting settings.

The main contributions of our paper are summarized as follows:

- We thoroughly investigate the features of siRNA and mRNA, including non-empirical and empirical rule-based features. This comprehensive analysis enhances the understanding of factors critical to siRNA efficacy that previous studies overlooked.
- We introduce **siRNADiscovery**, a Graph Neural Network (GNN) framework that leverages all the extracted features of siRNA and mRNA for enhanced prediction accuracy. Additionally, we use Optuna for streamlined hyperparameter optimization, contributing to the model’s robustness and efficiency.
- We conduct experiments on commonly used datasets under our proposed evaluation settings, where siRNADiscovery achieves *state-of-the-art* performance. Additionally, we conduct *in vitro***wet lab** studies and also collect a dataset for external validation, where siRNADiscovery shows superior performance on both datasets compared with other tools across all metrics.
- Our model is designed to serve as a robust foundation for advancing the development of predictive models in siRNA-mediated gene silencing, enhancing both research and practical applications.

## Materials and methods

### Data sources

#### Public data

In this work, we collected an siRNA dataset with 2816 siRNA–mRNA pairs and related efficacy values from Massimo *et al.* [12], which came from the original studies of Huesken [6], Harborth [20], Ui-Tei [21], Vickers [22], and Khovorova [23]. We named this dataset ‘Dataset_HUVK’. Additionally, we utilized the Simone dataset [24], which comprised 322 siRNAs, to further validate our models.

#### In-house data

Our experiment protocol is shown in Supplementary Material. We conducted multiple trials for each siRNA and obtained silencing efficiencies for 298 siRNAs. Those demonstrating negative knockdown efficiency were removed from further consideration. From the remaining siRNAs, we eliminated those that exhibited significant variability across trials due to experimental errors. By computing the average knockdown efficacy across various trials within the same experimental setup, we refined our dataset to include 102 siRNAs with consistent results, forming our final validation set.

In Table 1, we summarize the characteristics of the three datasets utilized in our experiments, including the number of mRNAs, the number of siRNAs, and the count of siRNA–mRNA pairs.

**Table 1:** **Dataset Information.** We summarize the information of the three datasets utilized in our experiments, including the number of mRNAs, siRNAs, and siRNA–mRNA pairs.

Dataset Name	No. of siRNAs	No. of mRNAs	No. of Pairs
Dataset_HUVK [12]	2816	44	2816
Simone [24]	322	6	322
In-house(*ACON*)	102	1	102

### Dataset splitting

Prior works [8, 11, 12, 25] evaluated the models via 10-fold cross-validation. Specifically, each dataset was divided into ten equal parts, with nine parts used for training and the remaining one part for testing. This cycle was executed ten times, allowing each segment to serve as the test set once. However, we observed a data leakage issue within the cross-validation setting, i.e., an siRNA from the test set might have been included in the training phase, thereby compromising the integrity of the evaluation. Furthermore, existing methods [8, 11, 12, 25] employed the same subset for both validation and testing, which was neither reasonable nor practical for real-world applications. Consequently, we introduced a **new** evaluation setting. Here, we randomly divided the dataset into a training set, an evaluation set, and a test set with a ratio of 70:15:15. To avoid data leakage or deviation from randomness, we respectively treated siRNA and mRNA as the basis of division and repeated the splitting process 10 times with different random seeds. This approach yielded 20 distinct splits, offering a more robust and realistic evaluation. Note that we denoted the split datasets divided based on siRNA and mRNA as ‘siRNA-split data’ and ‘mRNA-split data’, respectively.

### Model structure

**Figure 1:**
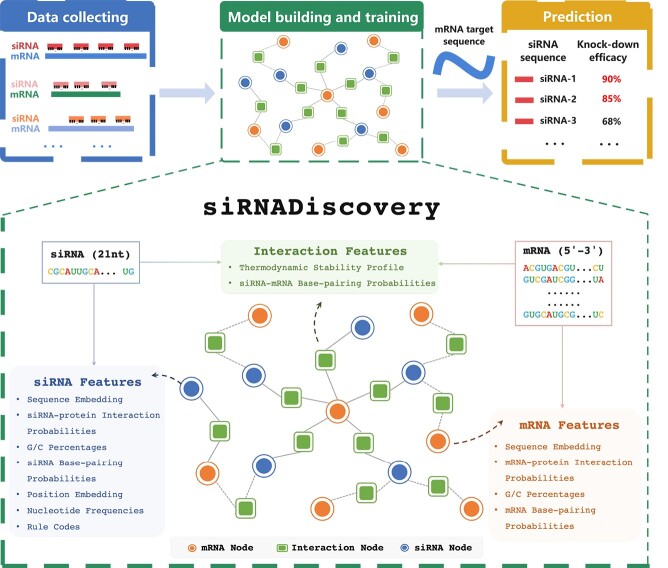
**Overall Workflow of siRNADiscovery.** Our model contains two modules, a **feature extraction module** for in-depth sequence characteristics exploration, and a **GNN prediction module** for feature fusion and efficacy regression.

As shown in Fig. 1, given the input siRNA and mRNA sequences, our model first extracts sequence characteristics comprehensively, and then assigns the features to the corresponding GNN nodes for feature fusion and efficacy regression. In the following paragraphs, we introduce the RNA sequence features and model structure in detail.

#### GNN Node Feature Assignment

To fully grasp the information embedded within mRNA and siRNA sequences, we encompassed the initial extraction of **two** types of features from the input RNA sequences and then assigned these features to the nodes of our GNN model. **The first type comprised non-empirical rule features**, which included sequence embeddings, positional embeddings, siRNA–mRNA base-pairing probabilities, and RNA–protein interaction probabilities. **The second type consisted of empirical rule features**, which are thermodynamic stability profile, nucleotide frequencies, G/C percentages, and siRNA rule codes. To further explore the intrinsic properties of mRNA/siRNA and their interaction, we employed advanced methodologies [26–29] to extract the aforementioned features. These methodologies collectively formed the feature extraction phase of our method.

Our model contains three types of nodes, mRNA nodes, siRNA nodes, and siRNA–mRNA interaction nodes, which are introduced in detail in Section 2.3.2. In Table 2, we show the features assigned to each kind of node, respectively. Below, we present the non-empirical rule features and empirical rule features in detail.

**Table 2:** **Assigned Features for Different Nodes.** We show the features assigned to each kind of node. ‘mRNA’, ‘siRNA’, and ‘Interaction’ represent the mRNA node, the siRNA node, and the siRNA–mRNA interaction node, respectively.

**Node Type**	**Assigned Features**
mRNA	Sequence Embedding
	mRNA–protein Interaction Probabilities
	G/C Percentages
	mRNA Base-pairing Probabilities
siRNA	Sequence Embedding
	siRNA–protein Interaction Probabilities
	G/C Percentages
	siRNA Base-pairing Probabilities
	Position Embedding
	Nucleotide Frequencies
	Rule Codes
Interaction	Thermodynamic Stability Profile
	siRNA–mRNA Base-pairing Probabilities

**For the non-empirical rule features**:

- ***Sequence Embedding.*** We employ one-hot encoding to represent biological sequences. Each nucleotide of the siRNA and mRNA sequences, corresponding to the four DNA bases A, C, G, and T, is encoded by a unique four-dimensional binary vector: A = <1, 0, 0, 0>, C = <0, 1, 0, 0>, G = <0, 0, 1, 0>, T = <0, 0, 0, 1>. For ambiguous nucleotides, we denote them as ‘N’ with a distinct vector <0, 0, 0, 0>.
- ***Positional Embedding of siRNA.*** The positional context of siRNA along the mRNA strand is crucial for its gene silencing efficacy [15]. Each position of siRNA along the related mRNA is encoded into a higher-dimensional space of dimension $D$, then a positional encoding matrix **P** of a dimension $n \times D$ is calculated in an element-wise manner. $\mathbf{P}_{ij} $ is defined as below:
  (1) \begin{align*} & \mathrm{P}_{i j}= \begin{cases} \ \mathit{sin} \left(\frac{\textstyle i}{\textstyle 10000^{\frac{\text 2 k}{D-1}}}\right), & \text{if}\ j=2 k \\[10pt] \ \mathit{cos} \left(\frac{\textstyle i}{\textstyle 10000^{\frac{\text 2 k}{D-1}}}\right), & \text{if}\ j=2 k+1, \end{cases}\end{align*}
  where $i$ denotes the position of siRNA on the mRNA strand and $j$ is the channel dimension.
- ***siRNA–mRNA Base-pairing Probabilities.*** Previous research has shown that the efficacy of RNA interference (RNAi) approaches is primarily determined by the sequence of siRNA or the structure of mRNA [30]. Both siRNA and mRNA strands are capable of folding into secondary structures independently, a process largely driven by nucleotide base pairing including canonical (A-U, C-G), non-Watson–Crick (G-U), and non-canonical interactions, which establish their secondary structures [31]. Studies have indicated that the base-pairing structure is more crucial for RNA’s proper function than the primary sequence itself in many cases [16]. Additionally, siRNA and mRNA could form complexes through intermolecular base pairing. Consequently, we used the RNAfold tool to obtain the base-pairing probability matrix for siRNA and mRNA, and RNAcofold for the siRNA–mRNA interaction base-pairing probability matrix. Both tools are part of the ViennaRNA package (Version 2.6.4) [26]. Due to the prevalence of zero probabilities in each matrix, truncated singular value decomposition (SVD) was employed to reduce dimensions and serve as a feature of the model.
- ***RNA–protein Interaction Probabilities.*** In the process of RISC assembly, the guide strand binds to the Argonaute protein AGO2 and directs RISC to its complementary target RNA, which is subsequently cleaved and degraded by the RNase activity within AGO2 [32]. RNA–protein interactions are significant in the RNAi process, a factor previously overlooked in earlier studies for siRNA efficacy prediction. RPISeq [27] is a machine learning-based tool designed to predict RNA–protein interactions. We utilized this tool to predict the interaction between siRNA–AGO2 and mRNA–AGO2, obtaining probabilities using RPISeq-RF as a feature of our model.

**For the empirical rule features**:

- ***Thermodynamic Stability Profile.*** The prevailing view has shown that the thermodynamic stability profile of the siRNA duplex strongly influences the efficacy of siRNA by reflecting a guide strand selection mechanism [33]. The thermodynamic stability profile of the siRNA antisense strand includes the calculation of Watson–Crick pair free energy ($\Delta G$) between every two adjacent nucleotides, the total energy of the siRNA, and the difference in energy at the 5’ and 3’ end of siRNA. All these computations followed the work [28].
- ***Nucleotide Frequencies.*** Certain short motifs influence the function of siRNA. For instance, sequences containing AAAA or UUUU are likely to be terminated by RNA polymerase III transcription, and CCCC or GGGG motifs may impact RNAi function by forming a nucleotide quartet [17]. Nucleotide frequency represents the count of each nucleotide within an siRNA sequence and it is widely adopted in existing research [9, 34, 35]. Given the constant length of siRNAs, we calculated the frequencies of 1-mer (A, U, C, G), 2-mer (e.g., AU, CG, etc.), 3-mer (e.g., AUC, UCG, etc.), 4-mer (e.g., AUCG, UCGA, etc.), and 5-mer (e.g., AUCGA, UCGUA, etc.) segments with 4, 16, 64, 256, and 1024 possible motifs, respectively.
- ***G/C Percentages.*** GC content is a critical factor in the efficacy of siRNAs. Low GC content leads to weak and non-specific binding; conversely, high GC content could hinder the unwinding of the siRNA duplex by the RISC complex and helicase [18]. Moreover, a previous study has indicated that lower GC content in both the global and local flanking regions of siRNA binding sites leads to siRNA inhibition. Thus, we computed the GC content of siRNA and mRNA sequences [33].
- ***Rule Codes of siRNA.*** Previous studies have shown that certain nucleotides at specific positions could either enhance or impair the function of siRNA. Initially, these effects were cataloged in the paper [19] and subsequently simplified by He *et al.* [29]. In the simplified rules proposed by He *et al.*, an encoding of 1 indicates a nucleotide’s preference for enhancing siRNA efficiency, whereas an encoding of -1 denotes a preference for reducing efficiency. If no rule specifies such a preference, the encoding defaults to 0. Here, we represented these rules using a unique three-dimensional binary vector: 1 = <0, 0, 1>, 0 = <0, 1, 0>, -1 = <1, 0, 0>.

#### GNN Model of siRNADiscovery

Based on the preliminaries of GNNs and Graph Convolutional Networks (GCNs) as illustrated in the Supplementary Materials, we modeled the graph of siRNADiscovery as an undirected heterogeneous graph with three distinct node types, i.e., siRNA, mRNA, and their interactions. We approached efficacy prediction as a node regression task, where outputs from the feature extraction module were aptly assigned to corresponding nodes. The strategy for assigning extracted features is detailed in Table 2. As shown in Fig. 1, $M_{j}, j \in [1, m]$ and $S_{i}, i \in [1, s]$ are involved within the constructed graph, and $m, s$ are the total number of mRNA and siRNA, respectively. Also, node $I^{M_{j}}_{S_{i}}$ appears in the graph, indicating known interaction features to the GNN module. Following [12], we adopt the Heterogeneous GraphSAGE platform [36], which utilizes a dual-layer structure to aggregate each node’s feature with features of its neighbors. For any given node $v$, we utilize $N(v)$ to represent its neighborhood, facilitating the aggregation process. The feature vector of node $v$ at layer $l$ is denoted by $h_{v}^{(l)}$, while $h_{N(v)}^{(l)}$ represents the aggregated feature vector from its neighborhood, the update rule for node $v$ is formulated as:


(2) \begin{align*} & h_{v}^{(l+1)} = \sigma\left(W^{(l)} \cdot \text{CONCAT}\left(h_{v}^{(l)}, h_{N(v)}^{(l)}\right)\right),\end{align*}

where $W^{(l)}$ is a layer-specific weight matrix, $\sigma $ is a non-linear activation function, and $\text{CONCAT}$ denotes the concatenation of the node’s current features with the aggregated neighborhood features.

### Hyperparameter tuning

To avoid overfitting, we employed Optuna [37], a Bayesian optimization library, for efficient hyperparameter tuning and benchmarking. Optuna is an advanced framework specifically designed to facilitate the automatic exploration of hyperparameter spaces, aiming to identify the most effective combinations of hyperparameters through a systematic approach. In our study, we performed 100 trials of Bayesian optimization, adopting the Pearson Correlation Coefficient (PCC) as the primary metric to be maximized.

### Model evaluation

To evaluate the efficacy of our model, we employed three key metrics: the Pearson Correlation Coefficient (PCC), the Spearman Correlation Coefficient (SPCC), and the Area Under the receiver operating characteristic Curve (AUC). Both PCC and SPCC served to measure the correlation between the actual and predicted efficacies, with PCC applied to continuous data and SPCC to ordinal rankings. The formulas for these coefficients are as follows:


(3) \begin{align*} & P C C=\frac{1}{n-1} \sum_{i=1}^{n}\left(\frac{X_{i}-\bar{X}}{\sigma_{X}}\right)\left(\frac{Y_{i}-\bar{Y}}{\sigma_{Y}}\right), \end{align*}

(4) \begin{align*} & SPCC=1-\frac{6 \sum d^{2}}{n\left(n^{2}-1\right)}, \end{align*}

where *X* and *Y* denote the predicted values and observed labels, and *n* represents their common size. *d* represents the difference between the *X* rank and *Y* rank for each pair of data. In addition to these correlation coefficients, the AUC metric is employed to measure the overall predictive performance of the model. AUC values range from 0 to 1, where higher values indicate better performance.

## Results

### Training settings and model settings

We adopted Optuna [37] to conduct hyperparameter tuning. We conducted a series of trials focusing on various parameters, including the sizes of HinSAGE layers, hop neighbor samples, batch sizes, dropout rates, dimensions of positional embeddings, and the dimensions of reduced matrices for both RNA base-pairing probabilities and siRNA–mRNA base-pairing probabilities. The final settings and configurations of siRNADiscovery on siRNA-split data are detailed in Table 3.

**Table 3:** **Training Hyperparameters and Model Hyperparameters of siRNADiscovery on siRNA-split data.** We list the best combination of the training and model hyperparameters among all trials. ‘Dim.’ denotes feature dimension.

**Name**	**Values**
***Training Hyperparameters***	
Batch Size	64
Learning Rate	1e-3
Loss Function	MSE
Epoch Number	26
***Model Hyperparameters***	
HinSAGE Layer Size	[64, 32]
Hop Neighbor Sample	[12, 6]
Dropout Rate	0.1
Dim. of Positional Embeddings	6
Dim. of mRNA Reduced Matrices	100
Dim. of siRNA Reduced Matrices	6
Dim. of Pair Reduced Matrices	50

### Performance

To verify the effectiveness of our proposed method, we compared the performance of siRNADiscovery with five previous works, namely, GNN4siRNA [12], DSIR [25], s-Biopredsi [8], i-Score [8], and a CNN model [11]. We obtained the predicted results of DSIR, s-Biopredsi, and i-Score from the i-Score website. In addition, we reproduced the results of the CNN model [11] according to the introduction and details in its paper.

#### Performance on Dataset_HUVK

**Figure 2:**
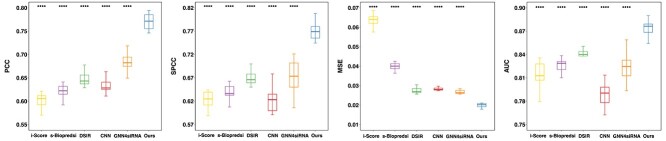
**Performance of siRNADiscovery on siRNA-split Data (Dataset_HUVK).** Our method achieves superior performance and remarkable stability across multiple metrics over other models. PCC: Pearson correlation coefficient; SPCC: Spearman correlation coefficient; AUC: Area Under Curve; MSE: Mean squared error. P values are calculated using the paired t-test to compare siRNADiscovery with each metric of other models. ^****^P$\leq 0.0001$.

We conducted experiments and reported the performances on siRNA-split data in Fig. 2. Experimental results under mRNA-split settings are presented in Supplementary Materials. As shown in Fig. S1, to avoid deviation from randomness, we conducted experiments under 10 distinct splits divided under different random seeds and report the average metrics and variance values. As shown in Fig. 2, our **siRNADiscovery outperforms the other five methods with small variation**, reaching an average PCC of 0.770, SPCC of 0.771, MSE of 0.020, and AUC of 0.874. Meanwhile, GNN4siRNA achieves an average PCC of 0.679, SPCC of 0.673, MSE of 0.026, and AUC of 0.821. Results of DSIR, s-Biopredsi, and CNN are worse than GNN4siRNA, while the i-Score model performs the worst with an average PCC, SPCC, and MSE of 0.602, 0.623, and 0.064, respectively.

#### Performance on Simone Dataset

**Figure 3:**
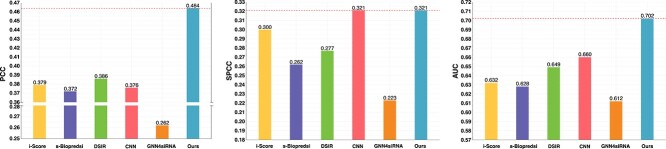
**Performance of siRNADiscovery on an External Dataset, Simone [24].** Notably, our siRNADiscovery shows a modest advantage over other methods, demonstrating the strong efficacy prediction capacity of our model.

Furthermore, we evaluated the effectiveness and robustness of these models on an external dataset, Simone [24], which contains 322 siRNAs. We trained all models on the training set of siRNA-split data and utilized the pre-trained models for inference on Simone. In Fig. 3, we show the evaluation results, where our siRNADiscovery has the best performance across metrics, achieving a PCC of 0.464 and an AUC of 0.702. However, the GNN4siRNA model’s predictions exhibit significantly lower performance, with a PCC of 0.262, SPCC of 0.223, and AUC of 0.612. This further demonstrates the superior robustness of our method even for external datasets compared with other methods.

#### Result Analysis for Dataset_HUVK and Simone

The unsatisfactory performance of DSIR, s-Biopredsi, and i-Score could be attributed to their reliance on traditional machine learning methods, which employ feature engineering and depend on empirical rules. Furthermore, important features like the location of the siRNA within the mRNA and the information about interactions between the siRNA and mRNA are absent from the GNN4siRNA model, hampering its capacity to identify underlying patterns among the features. Meanwhile, the inferiority of the CNN model may stem from a difference in focus. The convolutional kernels prioritize understanding local information, whereas comprehending RNA sequences requires an exploration of the entire sequence’s global content and meaning, as well as the overarching relationship between siRNA and mRNA.

#### Performance and Analysis on Our In-house Dataset

**Figure 4:**
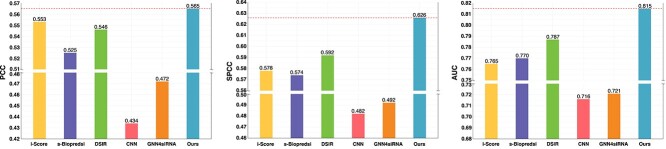
**Performance of siRNADiscovery on our In-house Dataset.** Notably, our siRNADiscovery shows a modest advantage over other methods, demonstrating the strong efficacy prediction capacity of our model.

We evaluated the robustness and generalization of all six models on our in-house dataset. As shown in Fig. 4, our siRNADiscovery outperforms the other models and achieves a PCC of 0.565, SPCC of 0.626, and AUC of 0.815.

**Figure 5:**
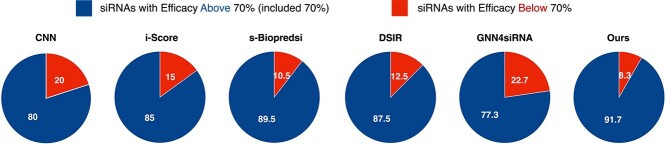
**Proportion of Top-200 siRNAs with Actual Efficacy Above/Below 70%.** We filter the siRNAs from various models based on their top-200 predicted values **trained on siRNA-split data**. We calculate and display the percentage of these siRNAs whose actual efficacy surpasses or falls below the 70% threshold.

**Figure 6:**
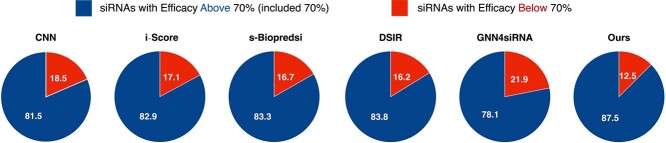
**Proportion of Top-300 siRNAs with Actual Efficacy Above/Below 70%.** We filter the siRNAs from various models based on their top-300 predicted values **trained on siRNA-split data**. We calculate and display the percentage of these siRNAs whose actual efficacy surpasses or falls below the 70% threshold.

Additionally, due to the limitation on the number of siRNAs, we selected the top-ranked siRNAs from the predictions of each method. Our siRNADiscovery has the highest proportion of siRNAs with actual efficacy above 70% (including 70%), reaching 91.7% in the top 200 and 87.5% in the top 300, respectively, as shown in Fig. 5 and Fig. 6.

### Ablation study

In our ablation study, we conducted a systematic evaluation to ascertain the impact of various features on performance metrics, specifically focusing on PCC across eight distinct categories of features on siRNA-split data. These categories include sequence embeddings, thermodynamic stability, interaction probabilities, RNA–protein interactions, positional embeddings, nucleotide frequencies, rule-based codes, and G/C content percentages. As illustrated in Table 4, the integration of all features yields the highest performance compared to other configurations. We observed a progressive enhancement in PCC as additional features were incrementally incorporated into the model. Notably, the inclusion of nucleotide frequencies leads to the most significant improvement, with a PCC increase of 0.03.

**Table 4:** **Ablation Study on Different Feature Components.** We report the PCC of siRNADiscovery on siRNA-split data. Clearly, the combination of all the proposed extracted features performs the best.

Sequence Embeddings	Thermodynamic Stability	Interaction Probabilities	RNA–protein Interaction	Positional Embeddings	Nucleotide Frequency	Rule Codes	G/C Percentages	PCC
✓	✓	–	–	–	–	–	–	0.731
✓	✓	✓	–	–	–	–	–	0.735
✓	✓	✓	✓	–	–	–	–	0.736
✓	✓	✓	✓	✓	–	–	–	0.738
✓	✓	✓	✓	✓	✓	–	–	0.768
✓	✓	✓	✓	✓	✓	✓	–	0.769
**✓**	**✓**	**✓**	**✓**	**✓**	**✓**	**✓**	**✓**	**0.770**

## Discussion

In this section, we delve into further discussions on our design choices, explorations, and directions for future research.

*****Performance on mRNA-split Data***** We conducted a rigorous evaluation on mRNA-split data through grid searches and ablation studies, where our model displayed comparable performance to several existing methods and surpassed that of both CNN [11] and GNN algorithms [12]. The results, shown in Fig. S1, reveal no significant performance disparity between our model and established methods such as DSIR [25] or s-Biopredsi [8], all of which outperform CNN and GNN4siRNA. Additionally, siRNADiscovery achieves a modest advantage on both Simone and in-house datasets, as illustrated from Fig. S2 to Fig. S5. Notably, our model maintains consistent stability across both siRNA-split and mRNA-split data.

The data leakage issue was observed in previous evaluations, where siRNAs from the test set might inadvertently be included in the training phase, thus undermining the evaluation’s integrity. We critiqued the impracticality of employing the same subset for both validation and testing as seen in existing methods. To counter this issue, we proposed and released new data-splitting methodologies based on siRNA and mRNA, which was previously unconsidered by researchers. This novel approach aims to set a new benchmark for future research, encouraging more rigorous and realistic evaluations.

*****Feature Engineering and Embedding Techniques***** Feature engineering plays a pivotal role in enhancing the performance of our siRNA efficacy prediction model. A key aspect of this process is the choice of sequence embedding techniques. We adopted one-hot encoding for sequence embedding rather than k-mers. As shown in Fig. S6, one-hot encoding consistently outperforms k-mers, which are utilized in the same manner as in GNN4siRNA, across various performance metrics. First, one-hot encoding provides precise and fine-grained positional information, with each nucleotide position individually encoded. In contrast, k-mers emphasize the local pattern and context within the sequence, potentially overlooking specific positional information of individual nucleotides due to the selection of k-values. Second, one-hot encoding transforms RNA sequences into two-dimensional binary matrices, offering an approachable way for them to be processed by deep learning models. The features generated by k-mers potentially lead to a significant increase in the feature space.

In addition to sequence embedding, we incorporated positional embeddings to capture the influence of siRNA target site location on mRNA. Studies [38, 39] have shown that the location of siRNA target sites on the mRNA significantly affects gene silencing efficiency. They demonstrate that siRNAs exhibit higher mismatch tolerance at 3’-UTR locations and greater repression in coding regions when targeted regions are structurally stable and require unraveling during translation processes [39]. Some existing methods have considered siRNAs’ positional effects but are limited to simplistic one-hot encoding strategies or sparse-semantic feature matrices with low dimensions. Our model’s high-dimensional positional embeddings provide better semantics for the learning process, enabling the GNN model to capture intricate sequence variations.

Furthermore, we integrated RNA base-pairing probabilities by incorporating siRNA–mRNA co-fold binding matrices and self-fold binding matrices for both siRNA and mRNA. These matrices capture the intrinsic and mutual interactions influencing silencing efficiency. Future enhancements may involve using CNNs for dimensionality reduction or seamlessly integrating these features into the model.

The RNA–AGO2 interaction features represent another crucial inclusion to improve siRNA efficacy prediction. The interaction between RNA and AGO2 is fundamental to the siRNA knockdown process. By integrating RNA–AGO2 interaction features, our model grasps a deeper understanding of the biological interactions at play. Future work could explore molecular docking techniques to assess binding affinities between nucleic acids and proteins, which may further refine siRNA knockdown predictions.

Finally, empirical rules derived from nucleotide frequencies play a significant role in improving our model’s predictive power. Our ablation studies show that siRNA nucleotide frequencies from 1-mer to 5-mer scales result in improved performance, corroborating prior research [9, 34, 35]. One possible reason for this influence is that nucleotide combinations contribute to RNA interference processes, such as influencing RNA secondary structure formation and complementary pairing with target mRNA, although further evidence is needed to confirm these effects. Moreover, this multi-scale feature representation, from individual nucleotides to complex multinucleotide combinations, enables the capture of dynamic changes and intricate local patterns within sequences, thereby providing a comprehensive understanding of sequence dependencies and variations.

*****In-depth Analysis on Why siRNADiscovery is Better***** Many existing methods, despite using advanced techniques like CNNs, fall short in capturing the complex, non-linear interactions between siRNA and mRNA, which are critical for effective gene silencing. While GNN models, such as GNN4siRNA, improve upon CNNs by leveraging graph structures to model siRNA–mRNA interactions, they still fail to incorporate key biological insights, such as positional context and empirical rules (e.g., thermodynamic stability and nucleotide frequencies). Moreover, their reliance on k-mer embedding introduces unnecessary noise, making it harder to accurately represent biological processes.

In contrast, siRNADiscovery integrates both non-empirical features (sequence encoding, base-pairing probabilities) and empirical features (thermodynamic profiles, rule codes) within a GNN framework. This combination allows the model to capture a more holistic view of siRNA–mRNA interactions, effectively addressing both intrinsic and extrinsic factors that influence silencing efficiency. The graph-based nature of GNNs is ideally suited for representing biological processes, where nodes and edges mirror the components and their relationships, enabling siRNADiscovery to model the complexity of siRNA–mRNA interactions with greater precision and less noise [40].

By treating these interactions as graphs, siRNADiscovery leverages the strengths of GNNs to capture nuanced patterns in gene regulation and expression, surpassing the limitations of previous models. Our model’s architecture is specifically designed to exploit these graph structures, which explains its superior generalization across multiple datasets, consistently outperforming CNN-based methods and GNN4siRNA. Additionally, our ablation studies provide insights into how each feature contributes to the overall performance of siRNADiscovery. The integration of these diverse features within the GNN framework ensures that siRNADiscovery captures the full complexity of biological interactions, explaining its superior efficacy compared to earlier models.

**Directions for Future Work** In the future, we plan to explore the chemical modifications of siRNAs and the integration of transformer models and other advanced deep-learning architectures, such as Graph Attention Networks. These avenues hold the promise of further enhancing the predictive accuracy and understanding of siRNA efficacy. Additionally, in our siRNADiscovery, we utilize NCBI BLAST[41] to screen for sequences with low off-target effects, and we are planning to develop additional methods to enhance this screening, which is crucial for the safe and effective therapeutic application of siRNAs.

## Conclusion

In this paper, we introduce **siRNADiscovery**, a Graph Neural Network (GNN) framework that marks a significant advancement in the prediction of siRNA efficacy. To alleviate the limitations observed in previous models [12] that lack a comprehensive understanding of the siRNA silencing mechanism, our model delves deeply into both the non-empirical and empirical rule-based features of siRNA and mRNA sequences. In this way, our siRNADiscovery comprehensively captures the intricate features and semantics of gene silencing and achieves *state-of-the-art* results on internal datasets. Significantly, siRNADiscovery also demonstrates superior performance compared to current methodologies in both *in vitro* wet lab experiments and on an externally collected dataset. Moreover, to address the critical issue of data leakage and the impracticality of conventional validation methods, we propose an innovative dataset-splitting methodology that enhances the evaluation’s integrity and realism. This methodology, by treating siRNA and mRNA as separate entities for dataset division, ensures a robust and unbiased assessment of model performance across various datasets. Extensive experiments, encompassing widely used and external datasets, demonstrate the superiority of our **siRNADiscovery**, showcasing unparalleled predictive performance and robustness across different experimental settings. We aim for our model to offer a solid foundation for developing more reliable predictive models in the field of gene silencing, and for our proposed dataset-splitting methodology to set a new benchmark for future research, encouraging more rigorous and realistic evaluations.

**Key Points**

- We thoroughly investigate the features of siRNA and mRNA, including non-empirical and empirical rule-based features. This comprehensive analysis enhances the understanding of factors critical to siRNA efficacy that previous studies overlooked.
- We introduce **siRNADiscovery**, a Graph Neural Network (GNN) framework that leverages all the extracted features of siRNA and mRNA for enhanced prediction accuracy. Additionally, we use Optuna for streamlined hyperparameter optimization, contributing to the model’s robustness and efficiency.
- We conduct experiments on commonly used datasets under our proposed evaluation settings, where siRNADiscovery achieves *state-of-the-art* performance. Additionally, we conduct *in vitro***wet lab** studies and also collect a dataset for external validation, where siRNADiscovery shows superior performance on both datasets compared with other tools across all metrics.
- Our model is designed to serve as a robust foundation for advancing the development of predictive models in siRNA-mediated gene silencing, enhancing both research and practical applications.

## Supplementary Material

Supplementary_Materials_bbae563

## Data Availability

The code supporting this study is publicly available at https://github.com/BertramLoong/siRNADiscovery. In-house data used in this article will be shared upon reasonable request to the corresponding author.
