# Supplementary material for: siRNADiscovery: a graph neural network for siRNA efficacy prediction via deep RNA sequence analysis
Source: Brief Bioinform. 2024 Nov 6;25(6):bbae563. doi: 10.1093/bib/bbae563 (PMC11539000; doi:10.1093/bib/bbae563)
Supplement: Supplementary_Materials_bbae563 [file supplementary_materials_bbae563.pdf]

Overview

- Section B: Knockdown of target genes by siRNA in vitro
- Section C: Preliminaries of GNNs and GCNs
- Section D: Experiments on mRNA-split Data.
- Section E: Comparison of one-hot encoding and k-mers as feature representations.
- Section F: Ablation Study on mRNA-split Data.
- Section G: Comparison of siRNA position distribution across prediction models.
- Section H: Visualizations of Hyperparameter Tuning.
- Section I:Probability Distribution of Sequence Similarity.
- Section J: Model performance relative to maximum sequence similarity between test and training sets.
- Section K: Comprehensive Ablation Study on Feature Importance

Knockdown of target genes by siRNA in vitro

We conducted *in vitro* wet lab studies, and the collecting procedures were followed to test the silencing efficiency of the designed siRNA on the target SERPINC1. In detail, on day 0, 293A cells (ATCC, Manassa, VA, US) were cultured in a 96-well cell culture plate with 100 μL culture medium. The initial seeding was 12,000 cells per well. On day 2, the medium was replaced with 150 μL. 0.4 μL of Lipofectamine 2000 (Thermo Fisher Scientific, Waltham, MA, US) was used as a plasmid transfection agent for the cells. Four different concentrations (10 nM initial, 10-fold dilution) of designed siRNA (in-house synthesized by ACON Pharmaceuticals, Cranbury, NJ, US), the negative control wells (transfection with luciferase plasmid only) and the background control wells (wells containing cells) were used. On day 2, after 24-hour transfection, double luciferase detection was performed, and silencing efficiency was calculated accordingly.

Preliminaries: GNNs and GCNs

In this section, we delved into the design and utility of classical Graph Neural Networks (GNNs) and Graph Convolutional Networks (GCNs), which form the foundational elements of our model tailored for processing graph-structured data.

Unlike conventional machine learning models that operate in Euclidean spaces, GNNs are uniquely adept at managing data that naturally forms networks or graphs. This is particularly relevant in the biological research sphere, where complex systems like protein interactions and metabolic pathways are often conceptualized as networks. GNNs mathematically represent graphs as $G = (V, E)$, with V denoting vertices or nodes, and E representing edges where each edge $e_{ij} \in E$ connects nodes v_i and v_j. The structure of a graph is further detailed by its adjacency matrix $A \in \mathbb{R}^{n\times n}$, while node and edge attributes are depicted through feature matrices $X_v \in \mathbb{R}^{n\times d}$ and $X_e \in \mathbb{R}^{e\times c}$, accommodating heterogeneity among nodes with distinct features.

Building upon the GNN framework, GCNs integrate the convolutional concept traditionally used in image processing to enhance the analysis of graph-structured data. By aggregating feature information from a node's local neighborhood through the adjacency matrix A and the node and edge feature matrices X_v and X_e, GCNs capture the topological structure of the graph. This aggregation enables GCNs to learn node representations that incorporate both their features and the influences of their adjacent nodes. The capability of GCNs to encode both node and structural information into dense vector representations proves highly effective for tasks such as node classification, link prediction, and graph classification, where spatial relationships among data points are critical.

Experiments on mRNA-split Data

In this section, we presented the additional experiments of our method on mRNA-split data, besides the siRNA-split data in the main paper.

Training Settings and Model Settings

Similar to the experiments on siRNA-split data in the main paper, we adopted Optuna [2] to conduct hyperparameter tuning encompassing a series of trials focusing on various parameters. We list the final settings and configurations of siRNADiscovery on mRNA-split data in Table S1.

Name	Values
Training Hyperparameters	
Batch Size	64
Learning Rate	1e-3
Loss Function	MSE
Epoch Number	38
Model Hyperparameters	
HinSAGE Layer Size	[64, 32]
Hop Neighbor Sample	[4, 2]
Dropout Rate	0.3
Dim. of Positional Embeddings	3
Dim. of mRNA Reduced Matrices	500
Dim. of siRNA Reduced Matrices	15
Dim. of Pair Reduced Matrices	50

Table S1. Training Hyperparameters and Model Hyperparameters of siRNADiscovery on mRNA-split data. We list the best combination of the training and model hyperparameters among all trails. 'Dim.' denotes feature dimension.

Performance

To further verify the effectiveness of our proposed method, we presented more results of siRNADiscovery compared with five previous works, i.e., GNN4siRNA [3], DSIR [4], s-Biopredsi [5], i-Score [5] and a CNN model [6], on Dataset_HUVK and two external datasets.

Performance on Dataset_HUVK

We conducted experiments and reported the performances on mRNA-split data in Figure S1. To avoid deviation from randomness, we conducted experiments under 10 distinct splits divided under different random seeds and reported the average metrics and variance values. As shown in Figure S1, our siRNADiscovery achieves comparable performance with small variation.

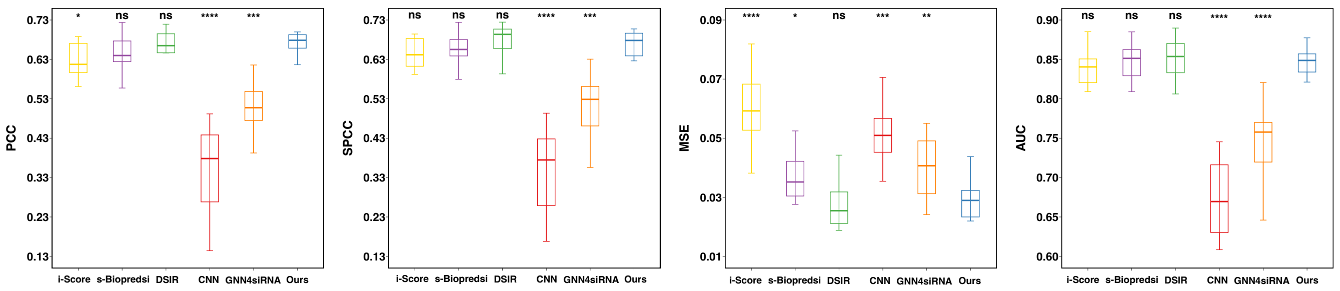

Figure S1: **Performance of siRNADiscovery on mRNA-split Data (Dataset_HUVK).** Our method achieves comparable performance and remarkable stability across multiple metrics over other models. PCC: Pearson correlation coefficient; SPCC: Spearman correlation coefficient; AUC: Area Under Curve; MSE: Mean squared error. P values are calculated using paired t-test to compare the siRNADiscovery with the metric of other models. $* * * * P \leq 0.0001$. $* * * P \leq 0.001$. $* P \leq 0.05$. $ns\ P \geq 0.05$.

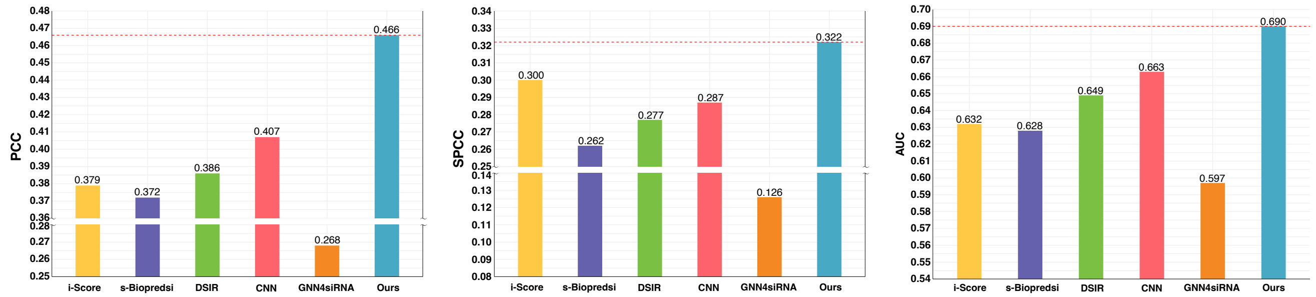

Figure S2: **Performance of siRNADiscovery on an External Dataset, i.e., Simone [1].** Notably, our siRNADiscovery surpasses other methods, demonstrating the strong efficacy prediction capacity of our model.

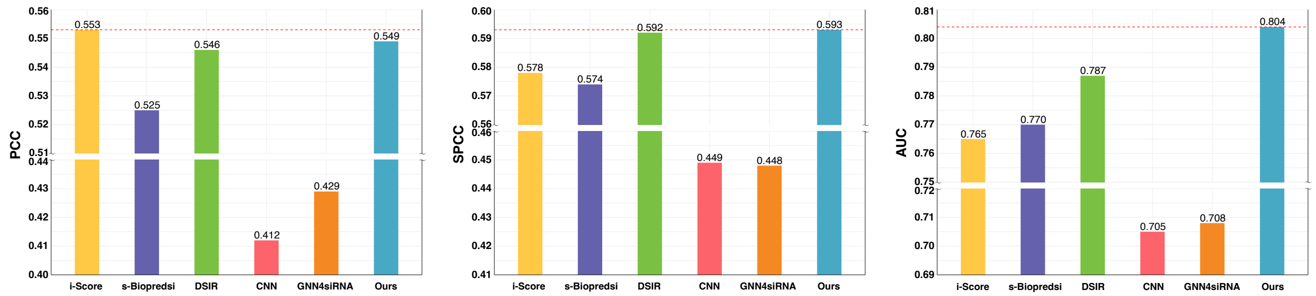

Figure S3: **Performance of siRNADiscovery on our In-house Dataset.** Notably, our siRNADiscovery shows a modest advantage over many methods, demonstrating the strong efficacy prediction capacity of our model.

Performance on Simone Dataset

Moreover, we evaluated the effectiveness and robustness of these models on an external dataset, Simone [1], which contains 322 siRNAs. Different from the experiments in the main paper, we first trained all models on the training set of **mRNA-split** data and utilized the pre-trained models to conduct inference on Simone. In Figure S2, we present the evaluation results, where our siRNADiscovery shows the best performance across all metrics, achieving a PCC of 0.466, SPCC of 0.322, and AUC of 0.690. This further demonstrates the superior robustness of our method, even for external datasets compared with other methods.

Performance on Our In-house Dataset

We assessed the robustness and generalization capabilities of all six models using our in-house dataset. As illustrated in Figure S3, our siRNADiscovery model surpasses the other models in terms of SPCC and AUC, achieving an SPCC of 0.593 and an AUC of 0.804. Additionally, our model achieves the second highest PPC at 0.549, narrowly trailing behind the leading model, i-Score [5], by only 0.004. This demonstrates the competitive performance of our siRNADiscovery model on mRNA-split data as well.

Similarly to the evaluation on siRNA-split data, we selected top-ranked siRNAs from predictions of each method. We noticed that our siRNADiscovery has a competitive proportion of siRNAs with

Sequence Embeddings	Thermodynamic Stability	Interaction Probabilities	RNA-protein Interaction	Positional Embeddings	Nucleotide Frequency	Rule Codes	G/C Percentages	PCC
✓	✓	-	-	-	-	-	-	0.646
✓	✓	✓	-	-	-	-	-	0.652
✓	✓	✓	✓	-	-	-	-	0.652
✓	✓	✓	✓	✓	-	-	-	0.654
✓	✓	✓	✓	✓	✓	-	-	0.662
✓	✓	✓	✓	✓	✓	✓	-	0.669
✓	✓	✓	✓	✓	✓	✓	✓	**0.672**

Table S2. Ablation Study on Different Feature Components on mRNA-split Data. We report the PCC of siRNADiscovery on siRNA-split Data. Clearly, the combination of all the proposed extracted features performs the best.

truth efficacy above 70% (included 70%), reaching 88.9% in the top 200 and 87.9% in the top 300, respectively shown in Figure S4 and Figure S5.

Comparison of one-hot encoding and k-mers as feature representations

In this section, we compared the performance of one-hot encoding and k-mers as feature representations in model training. As shown in Figure S6, the performance metrics demonstrate that one-hot encoding substantially surpasses k-mers when implemented as a training feature.

Ablation Study on mRNA-split Data

In this section, we extended our systematic evaluation to examine the influence of various feature categories on performance metrics, focusing on the PCC using mRNA-split data. The evaluated feature categories include sequence embeddings, thermodynamic stability, interaction probabilities, RNA-protein interactions, positional embeddings, nucleotide frequencies, rule-based codes, and G/C content percentages. As depicted in Table S2, the cumulative integration of these features leads to the highest performance relative to other configurations. A gradual improvement in PCC is observed as features were progressively added to the model.

Comparison of siRNA position distribution across prediction models

In this section, we compared the distribution of the top 200 and top 300 siRNA positions predicted by each method for SERPINC1 target. As shown in Figure S7 the siRNADiscovery algorithm shows a more uniform and broad distribution in predicting siRNA positions, suggesting its potential for comprehensively identifying effective siRNAs.

Visualizations of Hyperparameter Tuning.

In this section, we visualized the optimization history and parallel coordinate plot that illustrated the relationships and patterns across multiple variables, allowing for an in-depth analysis of trends and anomalies in the datasets.

Optimization History

In Figure S8, we present the optimization history of our siRNADiscovery and GNN4siRNA [3] model (a) using Optuna [2], a sophisticated hyperparameter optimization framework. Subfigures (b) and (c) depict the optimization trajectories on both mRNA-split and siRNA-split datasets, highlighting the performance and robustness of our models. Notably, subfigures (b) and (c), which represent our siRNADiscovery model, show a more concentrated distribution of data points. This clustering indicates that our model not only achieved more consistent performance across different datasets but also exhibited superior robustness. Furthermore, the density of optimal points in these subfigures is significantly higher, suggesting that our model is capable of reliably reaching peak performance levels, underscoring its effectiveness in siRNA design. This visual analysis clearly demonstrates the practical advantages of our model over existing approaches, enabled by the Optuna optimization engine.

Parallel Coordinate Plot

In Figure S9, we use parallel coordinate plots to visualize the optimization of our siRNADiscovery model on both mRNA-split and siRNA-split data, shown in subfigures (a) and (b). This visualization includes a range of trials examining hyperparameters such as the sizes of HinSAGE layers, hop neighbor samples, batch sizes, dropout rates, dimensions of positional embeddings, and dimensions of reduced matrices for both RNA base-pairing probabilities and siRNA-mRNA base-pairing probabilities. The plot indicates that our model's performance was highly sensitive to changes in batch size and epochs, suggesting that these parameters are pivotal for achieving optimal results. Conversely, the model demonstrates a lower sensitivity to other hyperparameters like dropout rates and dimensions of positional embeddings. This lower sensitivity could signify robustness in the model's architecture, implying that it could maintain effective performance under varying conditions of less critical parameters. Such robustness is advantageous in practical applications where varying data conditions and computational constraints require a model to perform consistently without extensive re-tuning of all parameters.

Probability Distribution of Sequence Similarity

In this section, we calculated the similarity between siRNAs in the test set and those in the training and validation sets using sequence similarity, defined as the proportion of matching nucleotides at the same positions in two sequences. In Figure S10, our results

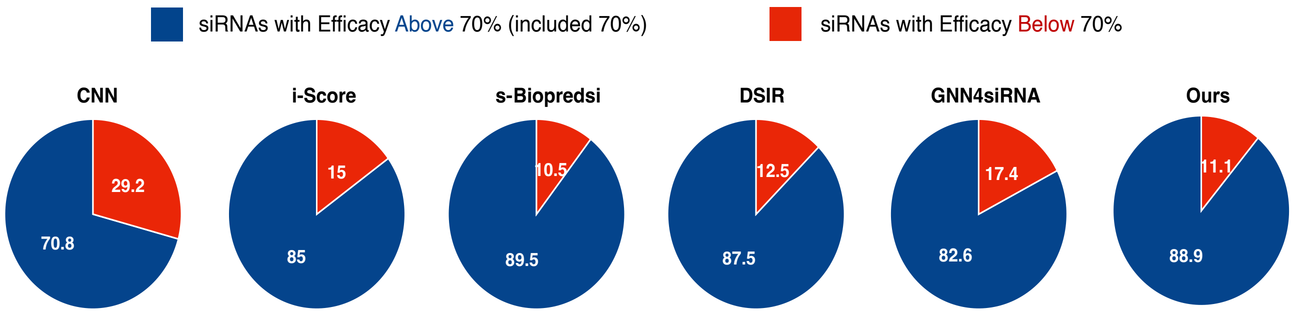

Figure S4: **Proportion of Top-200 siRNAs with Actual Efficacy Above/Below 70%.** We filter the siRNAs from various models based on their top-200 predicted values **trained on mRNA-split data**. We calculate and display the percentage of these siRNAs whose actual efficacy surpasses or falls below the 70% threshold.

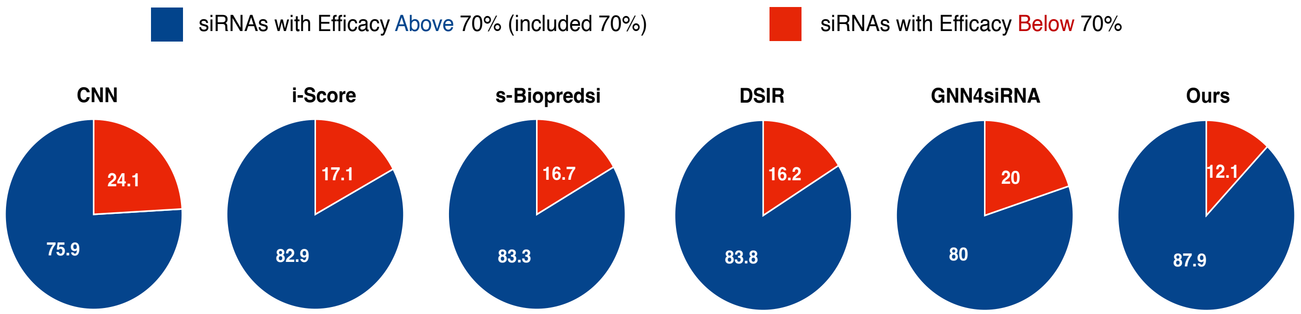

Figure S5: **Proportion of Top-300 siRNAs with Actual Efficacy Above/Below 70%.** We filter the siRNAs from various models based on their top-300 predicted values **trained on mRNA-split data**. We calculate and display the percentage of these siRNAs whose actual efficacy surpasses or falls below the 70% threshold.

show that the sequence similarity of the test set siRNAs with the training and validation sets remain below 80%, ensuring minimal overlap and demonstrating that our method does not overfit the training data. Additionally, we ensure that no identical siRNA sequences exist across or within training, validation, and test sets so that no siRNA-mRNA pairs are repeated, further reinforcing data independence.

The Relationship Between Maximum Similarity and Training-Test Set Differences

In Figure S11, we plot the siRNA-split data to illustrate the model's performance as a function of the maximum sequence similarity between the test and training sets. The Y-axis represents the absolute difference between actual and predicted knockout efficacy, while the X-axis corresponds to the maximum sequence similarity for each test set siRNA relative to the training set. The maximum sequence similarity for each siRNA in the test set is defined as the highest similarity value when compared with all siRNAs from the training sets. A linear regression analysis reveals a trend of improved prediction accuracy with increasing sample similarity. Notably, our method also achieves strong performance on lower similarity samples, indicating the model's robustness and generalization ability across diverse sequences. This result aligns with the typical behavior of deep networks, where samples closer to the training set are predicted more accurately, motivating our use of expert-driven feature design, robust training methods, and well-designed model structures to better capture underlying data patterns.

Comprehensive Ablation Study on Feature Importance

In this section, we conducted a comprehensive ablation analysis, as detailed in Tables S3 and S4. We systematically evaluate the effect of each feature by testing their individual addition to the model and assess the impact of their removal, replacing each removed feature with noise. This approach allows us to evaluate the contribution of each feature while maintaining a consistent input feature quantity, ensuring a fair comparison. The results reveal that while GC content and Rule codes exhibited a minor effect, they nonetheless enhanced prediction efficacy. In contrast, nucleic acid frequency emerges as the most significant factor affecting performance. Notably, the removal of any single feature consistently leads to a decline in model performance, underscoring the importance of each feature in the overall predictive framework.

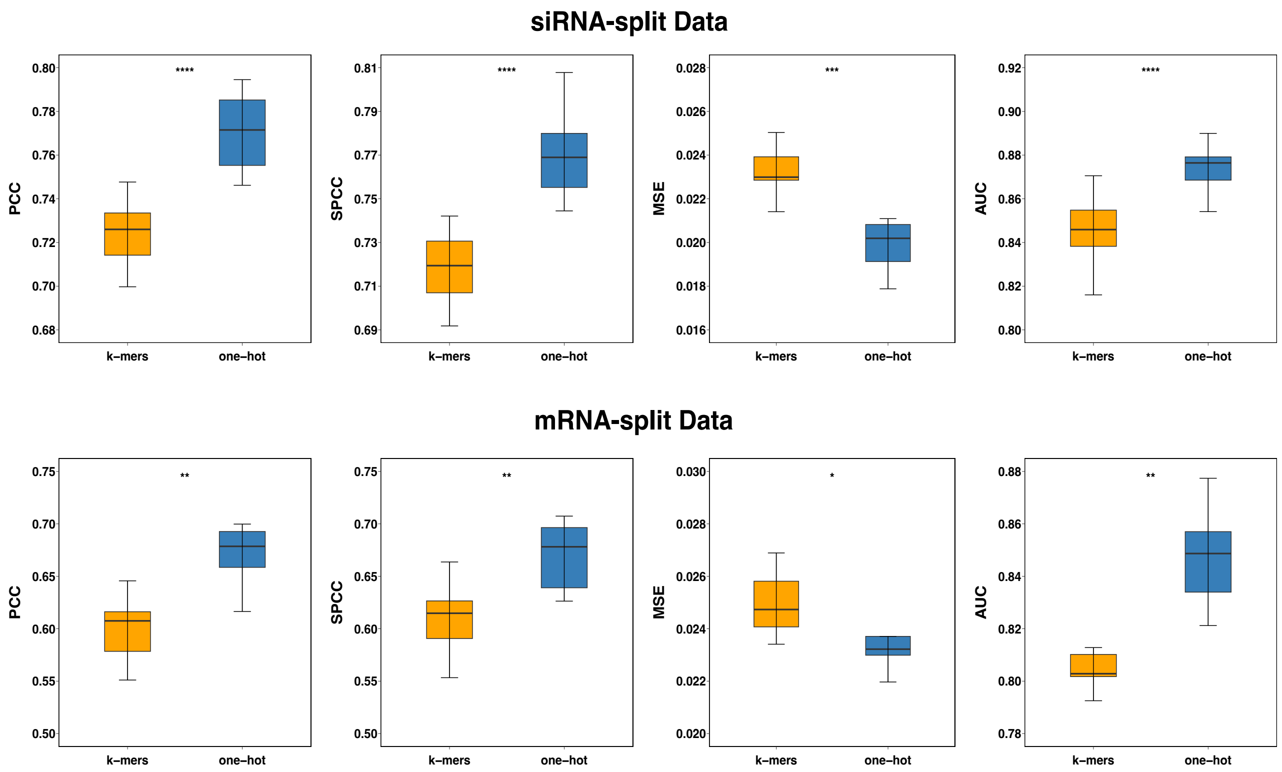

Figure S6: **Comparison of one-hot encoding and k-mers as feature representations.** The plots demonstrate that the performance metrics when using one-hot encoding as a training feature are significantly better than those when using k-mers. P values are calculated using paired t-test with the metric of other models. $****P \leq 0.0001$, $***P \leq 0.001$, $**\ P \leq 0.01$, $*\ P \leq 0.05$.

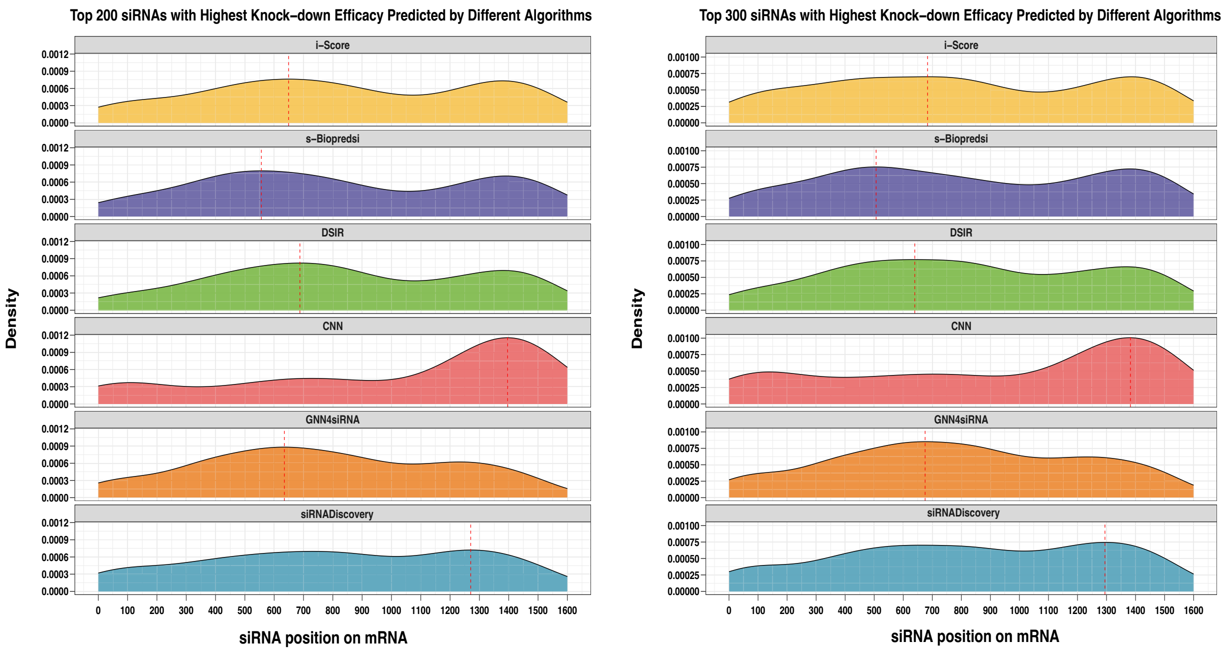

Figure S7: **Comparison of siRNA position distribution across prediction models.** Our siRNADiscovery exhibits a wider and more consistent spread in predicting siRNA positions on SERPINC1 target compared with other methods.

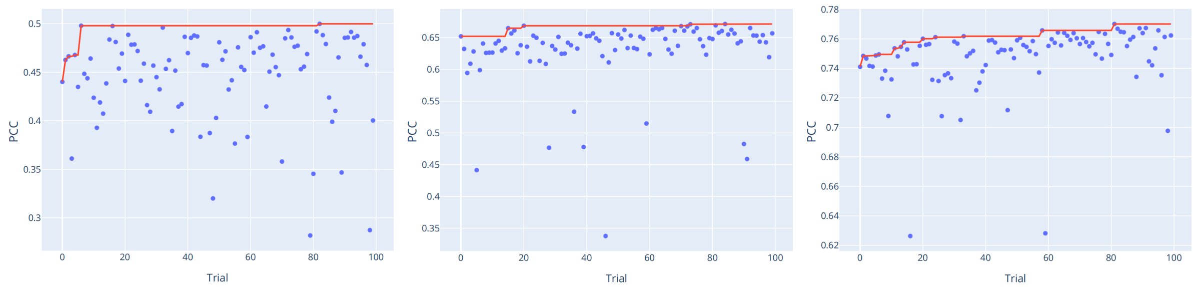

(a) **GNN4siRNA** (b) **siRNADiscovery** on mRNA-split Data (c) **siRNADiscovery** on siRNA-split

Figure S8: **Optimization history of siRNADiscovery and GNN4siRNA models using Optuna.** In subfigures (b) and (c), the concentration of data points indicates higher stability and robustness of the siRNADiscovery model, with a higher density of optimal points suggesting superior optimization outcomes. This visualization effectively demonstrates the enhanced performance and reliability of our approach in computational siRNA design.

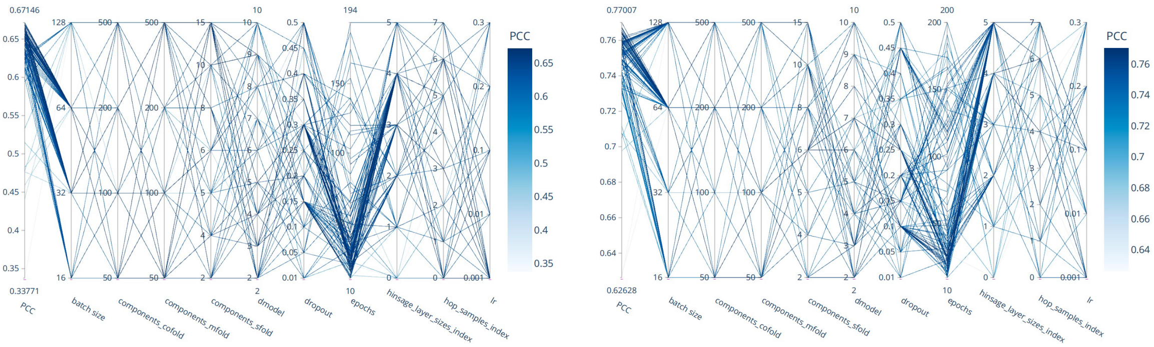

(a) siRNADiscovery on mRNA-split Data (b) siRNADiscovery on siRNA-split Data

Figure S9: **Hyperparameter sensitivity analysis of the siRNADiscovery model visualized through a Parallel Coordinate Plot.** Subfigures (a) and (b) illustrate the model's response to varying parameters on mRNA-split and siRNA-split data. The plot highlights the model's significant sensitivity to batch size and epochs, while showing robustness to variations in other parameters like dropout rates and embedding dimensions.

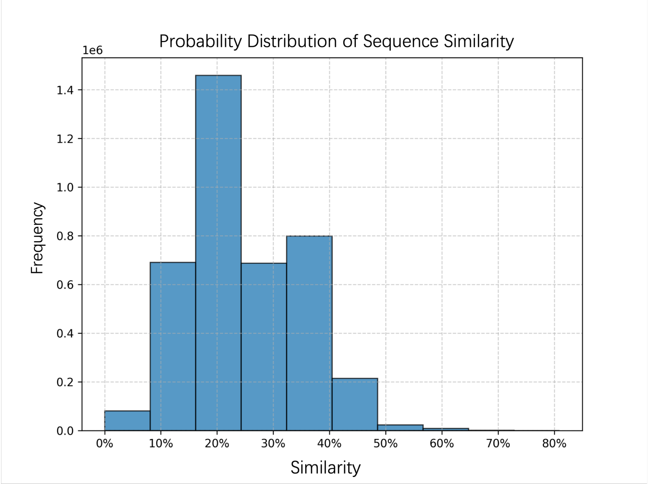

Figure S10: **The Probability Distribution of Sequence Similarity.** Distribution of sequence similarity between every two siRNAs within our Dataset_HUVK, showing that the similarity remained below 80%, indicating minimal overlap and reduced risk of overfitting. Sequence similarity is defined as the proportion of matching nucleotides at the same positions in two sequences.

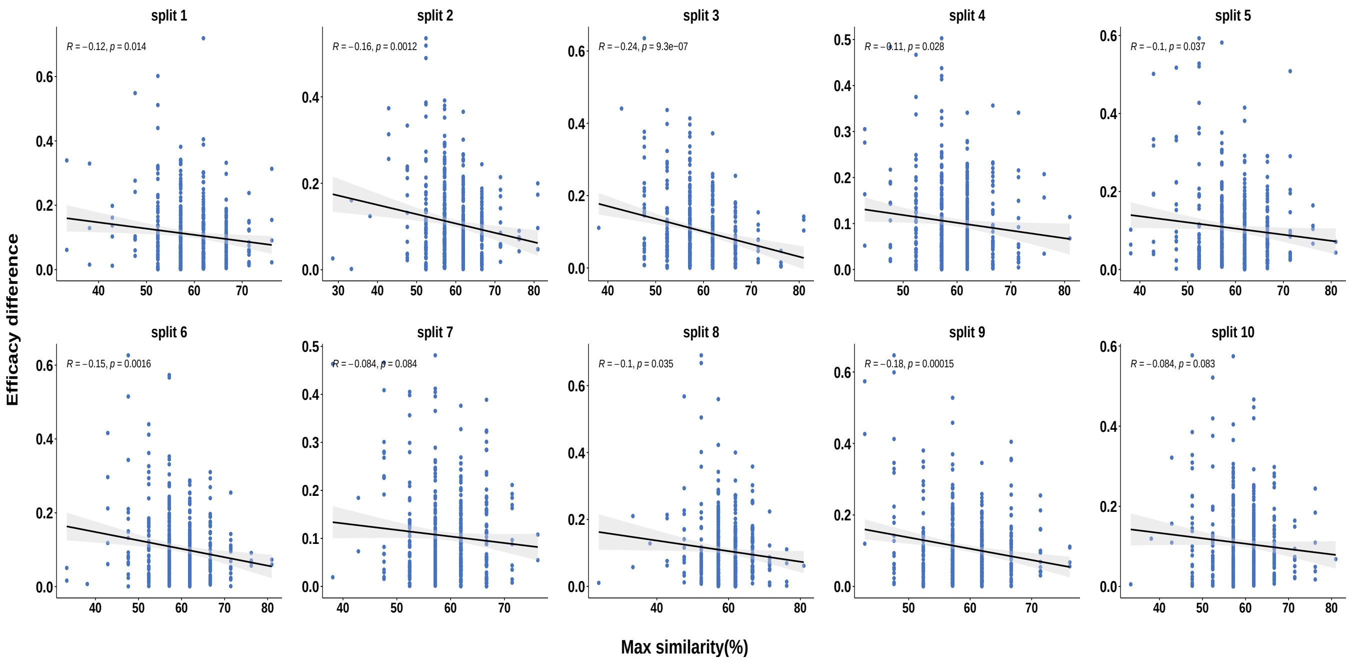

Figure S11: **Model performance relative to maximum sequence similarity between test and training sets.** Relationship between the absolute difference in knockout efficacy (Y-axis) and maximum sequence similarity between test and training sets (X-axis) on the siRNA-split data, highlighting the model's strong performance even on lower similarity samples, demonstrating its robustness and generalization ability. The maximum sequence similarity for each siRNA in the test set is defined as the highest similarity value when compared with all siRNAs from the training sets.

Interaction Probabilities	RNA-protein Interaction	Positional Embeddings	Nucleotide Frequency	Rule Codes	G/C Percentages	PCC
-	-	-	-	-	-	0.735
✓	-	-	-	-	-	0.741
-	✓	-	-	-	-	0.742
-	-	✓	-	-	-	0.750
-	-	-	✓	-	-	0.765
-	-	-	-	✓	-	0.741
-	-	-	-	-	✓	0.746

Table S3. Effect of Individual Feature Addition on Prediction Efficacy. We show the results of adding each feature individually on siRNA-split data, highlighting their contributions to prediction performance.

Sequence Embeddings	Thermodynamic Stability	Interaction Probabilities	RNA-protein Interaction	Positional Embeddings	Nucleotide Frequency	Rule Codes	G/C Percentages	PCC
✓	✓	✓	✓	✓	✓	✓	✓	0.770
✓	✓	✓	✓	✓	✓	✓	Noise	0.756
✓	✓	✓	✓	✓	✓	Noise	✓	0.757
✓	✓	✓	✓	✓	Noise	✓	✓	0.721
✓	✓	✓	✓	Noise	✓	✓	✓	0.749
✓	✓	✓	Noise	✓	✓	✓	✓	0.751
✓	✓	Noise	✓	✓	✓	✓	✓	0.740
✓	Noise	✓	✓	✓	✓	✓	✓	0.733

Table S4. Impact of Feature Removal with Noise Replacement on Prediction Efficacy. We present results from removing each feature and replacing it with noise on siRNA-split data, maintaining a consistent input count for fair comparison.

References

1. Simone Sciabola, Qing Cao, Modesto Orozco, Ignacio Faustino, and Robert V Stanton. Improved nucleic acid descriptors for siRNA efficacy prediction. *Nucleic Acids Research*, 41(3):1383–1394, 2013.
2. Takuya Akiba, Shotaro Sano, Toshihiko Yanase, Takeru Ohta, and Masanori Koyama. Optuna: A next-generation hyperparameter optimization framework. In *Proceedings of the 25th ACM SIGKDD international conference on knowledge discovery & data mining*, pages 2623–2631, 2019.
3. Massimo La Rosa, Antonino Fiannaca, Laura La Paglia, and Alfonso Urso. A Graph Neural Network Approach for the Analysis of siRNA-Target Biological Networks. *International Journal of Molecular Sciences*, 23(22):14211, 2022.
4. Odile Filhol, Delphine Ciais, Christian Lajaunie, Peggy Charbonnier, Nicolas Foveau, Jean-Philippe Vert, and Yves Vandenbrouck. DSIR: assessing the design of highly potent siRNA by testing a set of cancer-relevant target genes. *PloS One*, 7(10):e48057, 2012.
5. Masatoshi Ichihara, Yoshiki Murakumo, Akio Masuda, Toru Matsuura, Naoya Asai, Mayumi Jijiwa, Maki Ishida, Jun Shinmi, Hiroshi Yatsuya, Shanlou Qiao, et al. Thermodynamic instability of siRNA duplex is a prerequisite for dependable prediction of siRNA activities. *Nucleic Acids Research*, 35(18):e123, 2007.
6. Ye Han, Fei He, Yongbing Chen, Yuanning Liu, and Helong Yu. SiRNA silencing efficacy prediction based on a deep architecture. *BMC Genomics*, 19:59–65, 2018.
